# Draft Genome Sequence of *Escherichia coli* S51, a Chicken Isolate Harboring a Chromosomally Encoded *mcr-1* Gene

**DOI:** 10.1128/genomeA.00796-16

**Published:** 2016-08-04

**Authors:** Katrin Zurfluh, Taurai Tasara, Laurent Poirel, Patrice Nordmann, Roger Stephan

**Affiliations:** a Institute for Food Safety and Hygiene, Vetsuisse Faculty, University of Zürich, Zürich, Switzerland; b Emerging Antibiotic Resistance Unit, Medical and Molecular Microbiology, Department of Medicine, Faculty of Science, University of Fribourg, Fribourg, Switzerland; c HFR–Hôpital Cantonal, Fribourg, Switzerland

## Abstract

We present the draft genome of *Escherichia coli* S51, a colistin-resistant extended-spectrum β-lactamase-producing strain isolated in 2015 from raw chicken meat imported from Germany. Assembly and annotation of this draft genome resulted in a 4,994,918-bp chromosome and revealed a chromosomally encoded *mcr-1* gene responsible for the colistin resistance of the strain.

## GENOME ANNOUNCEMENT

The recent description of the plasmid-mediated colistin resistance gene, *mcr-1*, in strains isolated from food animals, food, and humans in China was a signal for an avalanche of retrospective studies investigating the presence of this specific gene (1). The *mcr-1* gene has been identified almost all over the world now, and the earliest evidence for its presence dates back to the 1980s (2). The wide spread of *mcr-1* and the finding that this resistance marker is often associated with multidrug resistant *Enterobacteriaceae*, e.g., extended-spectrum β-lactamase (ESBL)-producers or carbapenemase-producers, is of great concern (3). The *mcr-1* gene was so far associated with different plasmid replicon types such as IncI2, IncHI2, IncP, IncFIP, and IncX4 (1, 4–8).

In a recent study, we isolated an ESBL-producing *E. coli* (new MLST sequence type: allelic profile: 6-4-5-16-24-1-14) harboring the *mcr-1* gene from raw chicken meat imported from Germany (9). Despite repeated attempts, the *mcr-1* gene from this isolate could not be transferred by conjugation. Plasmid DNA from S51 was extracted and used in electroporation experiments. Again the transfer of the colistin resistance determinant was also not successful. Further hybridization experiments probing with an *mcr-1* fragment indicated the possible chromosomal integration of the *mcr-1* gene (data not shown). Therefore, genomic DNA was isolated from S51 and subjected to sequencing using Pacific Biosciences SMRT technology at the ChunLab at Seoul National University. The S51 genome was assembled *de novo* using the SMRT Analysis 2.3.0 software to a single chromosome of 4,994,918 bp in size with a G+C content of 50.7% and two unclosed plasmid sequences of approx. 93 kb and approx. 98 kb in size, respectively. Gene prediction was carried out using Glimmer 3.0.2 (10). Annotation was conducted based on homology searches against COG, SEED, and KEGG databases (11–13) and using the NCBI Prokaryotic Genome Annotation Pipeline (PGAP) (http://www.ncbi.nlm.nih.gov/genome/annotation_prok/).

The *mcr-1* gene in S51 is located at the right-hand extremity of an IS*Apl1* element, together with an 813-bp orf encoding a hypothetical protein with similarities to a PAP2 superfamily protein. This combination of the IS*Apl1* and the *mcr-1* cassette has been often described on *mcr-*1-harboring plasmids of diverse replicon types (14). The “IS*Aspl1-mcr-1-*cassette” was found to be located in the S51 chromosome between the gene encoding for the outer membrane protein E and for a glutamate-5-kinase, respectively. This IS*Aspl1-mcr-1-*cassette association on a chromosome further supports the hypothesis that IS*Aspl1* might be involved in *mcr-1* acquisition.

### Accession number(s).

Sequence and annotation data of the draft genome of *E. coli* strain S51 and two plasmids were deposited in the GenBank database with the accession numbers CP015995, CP015996, and CP015997.
